# “Noticing the Way that I’m Noticing Pain”: A Qualitative Analysis of Therapeutic Progression in Mindfulness-Oriented Recovery Enhancement for Patients with Lumbosacral Radicular Pain

**DOI:** 10.1007/s12671-026-02782-1

**Published:** 2026-03-06

**Authors:** Ryan S. Wexler, Wade Balsamo, Devon J. Fox, Danielle ZuZero, Anand Parikshak, Sophia Kwin, Jillian Ramirez, Austin R. Thompson, Hans L. Carlson, Thomas Kern, Scott D. Mist, Ryan Bradley, Heather Zwickey, Courtney K. Pickworth, Eric L. Garland

**Affiliations:** 1 Helfgott Research Institute, National University of Natural Medicine, Portland, United States; 2 Department of Anesthesiology, University of Michigan–Ann Arbor, Ann Arbor, United States; 3 Center for Yoga Studies, Himalayan Institute, Honesdale, United States; 4 Yoga Therapy Program, The Mindfulness Center, Bethesda, United States; 5 Department of Orthopaedics and Rehabilitation, Oregon Health & Science University, Portland, United States; 6 Comprehensive Pain Center, Oregon Health & Science University, Portland, United States; 7 Herbert Wertheim School of Public Health and Human Longevity Sciences, University of California, San Diego, San Diego, United States; 8 Department of Psychiatry, University of California, San Diego, San Diego, United States; 9 Sanford Institute for Empathy and Compassion, University of California, San Diego, San Diego, United States

**Keywords:** Back pain, Radiculopathy, Mindfulness, Non-pharmacologic management, Qualitative analysis, Longitudinal analysis

## Abstract

**Objectives:**

Mindfulness-Oriented Recovery Enhancement (MORE) aims to foster adaptive attention and pain reappraisal. While research has demonstrated MORE’s efficacy, the progression through which participants modify their relationship to pain remains unclear. To understand this process, we conducted a qualitative study with data from a clinical trial of MORE for patients with lumbosacral radicular pain (LRP).

**Method:**

Thirty session recordings from MORE were coded. Using thematic analysis, we examined therapeutic processing sessions to understand how participants learned and applied mindfulness to pain. Analysis focused on dialogue resulting from MORE’s model of processing, Phenomenology, Utilization, Reframing, Education, Reinforcement (PURER).

**Results:**

Four stages and one barrier were identified: (1) Pain Vigilance and Attention Dysregulation, (2) Attention Regulation and its application to Experiential Avoidance, (3) Metacognitive Awareness, and (4) Pain Reappraisal. In addition, some participants engaged in experiential avoidance by using their newfound attention skills to avoid their pain experience. PURER emerged as crucial in facilitating adaptive pain engagement.

**Conclusions:**

The four-stage progression pattern identified here — the Vigilance-Avoidance Metacognition-Reappraisal (VA-MR) framework — may help clinicians anticipate challenges in mindfulness training. This study illuminates how MORE participants develop an adaptive relationship with chronic pain, and while attention regulation skills are necessary, they may initially be used for avoidance. Therapeutic benefit appears to require guidance through these stages by a skilled therapist who can navigate initial avoidance tendencies. These findings offer an actionable model for assessing progress and tailoring MBIs to enhance therapeutic outcomes.

Chronic pain is a pervasive health issue affecting approximately 20% of adults in the United States (Yong et al., 2022). Among chronic pain conditions, lumbosacral radicular pain (LRP), commonly known as sciatica or spine-related leg pain, presents a particularly challenging clinical picture (Mahmutović et al., 2024; Schmid et al., 2023). LRP is characterized by pain, paresthesias, and motor/reflex abnormalities (Tarulli & Raynor, 2007). This condition not only causes significant physical discomfort but also contributes to decreased quality of life (Konstantinou et al., 2013).

Traditional approaches to managing LRP involve pharmacological interventions, including opioid medications, which carry risks of dependence and abuse (Vowles et al., 2015). Considering these challenges, there is a growing interest in non-pharmacological approaches including mind-body interventions for chronic pain management. Mindfulness-based interventions (MBIs) have shown promise, demonstrating efficacy in reducing pain severity and improving pain-related functioning (Hilton et al., 2017). While previous research has demonstrated the effectiveness of MBIs for chronic pain conditions (Arefian & Asgari-Mobarake, 2025; Cayoun & Shires, 2020a; Cayoun et al., 2020b; Hanley et al., 2023; Hilton et al., 2017; Wexler et al., 2024), less is known about the psychological mechanisms and therapeutic progression through which participants develop adaptive relationships with pain.

Mindfulness-Oriented Recovery Enhancement (MORE) represents a novel approach within the field of MBIs (Garland, 2024; Garland et al., 2022; Parisi et al., 2022; Wexler et al., 2024). MORE integrates mindfulness training with principles from cognitive-behavioral therapy and positive psychology to specifically target the dysregulation of reward processing and attentional bias towards pain-related cues that often characterize chronic pain conditions (Garland & Howard, 2013). The program aims to enhance participants’ ability to notice and savor natural rewards while simultaneously developing a more adaptive relationship with pain sensations. Prior full-scale randomized controlled trials (RCTs) have demonstrated MORE’s efficacy for treating heterogeneous forms of chronic pain and reducing opioid use. In the largest trial, MORE reduced opioid misuse by 45%, nearly tripling the effect of standard group therapy while significantly decreasing pain severity and pain-related functional interference at 9-month follow-up (Garland et al., 2022). A more recent early-stage randomized controlled trial examined MORE’s efficacy specifically for LRP symptoms. While quantitative outcomes demonstrate MORE’s positive impact on pain intensity, understanding if and how participants’ relationship with pain changes throughout the intervention requires qualitative investigation (Wexler et al., 2022, 2024).

A key component of MORE is its structured model of therapeutic processing, known as PURER: Phenomenology, Utilization, Reframing, Education/Expectancy, Reinforcement (Garland, 2013, 2024). In each session, a trained clinician uses the PURER approach to (1) process meditative experiences via inquiry into moment-to-moment phenomenology, (2) utilize meditative experiences to support coping with symptoms outside of the session, (3) reframe challenges that arise during meditation, (4) provide education about meditative phenomena and build positive therapeutic expectancy, and (5) provide positive reinforcement for engaging in mindfulness practice (Table 1). PURER draws from behavior change theory principles of selective reinforcement (i.e., strengthening adaptive attentional or behavioral responses) and successive approximation (i.e., gradually shaping complex skills through reinforcing incremental steps) to shape participant responses to meditation practice. This helps participants successfully apply mindfulness to pain relief and other therapeutic goals (e.g., emotion regulation, reduction of opioid use). This model guides participants through a process of exploring pain experiences, reappraising their relationship to pain, and reinforcing adaptive coping strategies. Despite the growing body of research supporting the efficacy of MORE for various chronic pain conditions, its specific impact on LRP and the psychological mechanisms through which it influences pain perception and pain management remain underexplored. Understanding the therapeutic progression fostered by MORE’s approach to therapeutic processing, PURER, is crucial for optimizing intervention delivery and supporting participants through common challenges.

**Table 1:** PURER steps and definitions

PURER step	Definition
Phenomenology	Therapists used open-ended but directive questions to elicit detailed descriptions of participants’ moment-to-moment experiences during mindfulness practices, with a focus on eliciting positive or therapeutic experiences. Participants were queried about the temporal sequence and unfolding of their meditative experiences. These inquiries focused on physical sensations, emotional responses, and cognitive processes (e.g., “What did you notice about your experience?” “Where did your attention go?”). This phenomenological data formed the foundation for subsequent components of the framework, particularly a starting point in discussion that could be followed by other participants
Utilization	Therapists actively incorporated participants’ reported experiences into teaching key concepts and skills for coping in everyday life. Participants were asked how they could use what they learned from the mindfulness practice session to address their symptoms in everyday life outside of the session
Reframing	Guided discussion of alternative interpretations of pain experiences was used to help patients reinterpret their pain experience. In addition, these interpretations were connected to the content of the MORE session. Therapists helped participants examine their automatic pain-related thoughts and develop more adaptive perspectives. Therapists also reframed the inevitable challenges that arise during meditation as the practice of mindfulness
Education/expectancy	Educational elements were systematically integrated to help participants understand the relationship between attention, pain perception, and emotional responses. This included explanation of relevant neurobiological mechanisms of pain and the psychological principles underlying mindfulness practices. Importantly, therapists aimed to build an expectation of therapeutic benefit in this step
Reinforcement	Therapists consistently acknowledged and reinforced participants’ efforts to engage with mindfulness practices and their emerging insights about pain management. This positive reinforcement aimed to strengthen pain coping strategies and maintain engagement with the intervention

Further, research suggests that mindfulness can reduce pain-related suffering (McCracken et al., 2007); however, the path to therapeutic benefit may not be linear, as research indicates that initial attempts at mindfulness practice may present challenges in implementation and understanding. In addition, prior work has revealed multiple complex interactions between mindfulness, pain catastrophizing, pain intensity, and fear-avoidance patterns. Schütze et al. (2010) previously showed that mindfulness acts as a moderator of the relationship between pain catastrophizing and fear-avoidance, and that mindfulness also moderates the relationship between pain catastrophizing and pain intensity (Wilson et al., 2023).

To explore the relationship between pain and its sequelae, we analyzed session recordings from participant groups in the MORE arm of a randomized controlled trial for LRP patients. Our analysis focused on how participants described their experiences with mindfulness practice, pain perception, and the application of MORE skills in daily life. This study sought to address critical gaps in our understanding of how MBIs facilitate therapeutic change in chronic pain conditions. We aimed to identify the process by which MORE encourages a shift in pain perception that is often reported among individuals suffering with pain-related disability due to LRP. We hypothesized the presence of distinct stages in participants’ progression through phenomenological inquiry that may elucidate the mechanisms through which mindfulness influences pain perception. Understanding how participants navigate and develop an adaptive relationship with pain could inform clinical practice and theoretical models of mindfulness-based pain management.

## Method

### Participants

This qualitative investigation analyzed recorded sessions from cohorts of 37 participants receiving MORE as part of a randomized controlled trial for LRP (Wexler et al., 2022, 2024). Briefly, eligibility criteria for the parent study included participants aged 18–65 years who either had a physician-confirmed LRP diagnosis or reported chronic LRP symptoms with the presence of neuropathic pain on screening (painDETECT scores > 15). Exclusion criteria included recent epidural steroid injections (< 3 months), recent surgical interventions for back pain or radicular pain (< 6 months), an existing mindfulness practice, concurrent cancer diagnosis, and unmanaged psychotic disorders.

### Procedures

We analyzed 30 audio recordings from the 8-week MORE intervention, each consisting of 2-hr group sessions following the manualized MORE for Pain protocol (Table 2; Garland, 2013; Wexler et al., 2022). To protect participant confidentiality, identifying information was removed from transcripts and participants were assigned pseudonyms. Audio recordings and anonymized transcripts were stored on encrypted servers accessible only to the research team. All MORE sessions followed a similar structure which included mindfulness of pain practice, practice debriefing, daily homework review, new psychoeducational content, experiential exercises, and a closing discussion. Recordings were transcribed verbatim using Whisper speech recognition software, with researchers manually verifying transcript accuracy. To reduce potential bias in analysis of participants’ progress through the program, transcripts were stripped of session numbers and randomized before qualitative coding began.

**Table 2:** MORE session topics

Session 1	What is Pain and Why Can Mindfulness Help?
Session 2	Automaticity in Chronic Pain
Session 3	Mindful Reappraisal
Session 4	Mindful Savoring
Session 5	Relationship between Pain and Unhealthy Coping Mechanisms
Session 6	Disrupting the Link between Stress and Pain
Session 7	Pain and Thought Suppression, Mindfulness to Meaning and Interdependence
Session 8	Review and Discussion of Maintaining a Mindfulness Practice

### Measures

The primary data source for this qualitative analysis consisted of audio recordings from MORE group sessions. Recordings captured therapist–participant dialogue during mindfulness practices, guided inquiry using the PURER framework, and group discussion of participants’ experiences applying mindfulness skills to pain in daily life. Transcripts served as the primary analytic material for thematic analysis. Although the present study focused on qualitative data, participants in the parent randomized controlled trial completed standardized measures of pain intensity and disability (e.g., visual analogue scale for pain, Oswestry Disability Index). These measures were used descriptively to contextualize qualitative findings but were not directly analyzed in the present study.

### Data Analyses

We employed thematic analysis throughout the qualitative coding process. At weekly meetings, RW and WB discussed transcript excerpts, allowing codes to arise from the data. A preliminary codebook was developed using terms from the MORE manual and MBI literature, such as “chronic pain,” “guided inquiry,” “mindful reappraisal,” and “attention regulation.” This initial codebook was applied to a sample of seven transcripts, which were coded independently by both coders. After each transcript was coded, consensus meetings were held to refine the codebook and code definitions. Coding was completed using the Delve software (Delve, 2025).

To maintain methodological rigor, we implemented multiple validation strategies. Consensus meetings allowed for discussions about divergent interpretations of the data. In addition, we actively sought and analyzed negative cases — instances where participant experiences deviated from emerging patterns — to ensure our analysis captured the full range of participant experiences. Coding memos were used to document analytical decisions and emerging interpretations. Data triangulation included comparison with quantitative outcomes from the parent study and consultation with MORE therapists to verify interpretations of participant-therapist interactions (Carter et al., 2014).

Once a refined version of the codebook had been developed and all transcripts were coded, codes were organized into themes, with supporting quotes cataloged for each theme. The research team then reviewed these themes against both the coded excerpts and the complete dataset to ensure comprehensive representation of participant experiences. The resulting analysis provides a detailed account of participant experiences and therapeutic progression patterns. While our analysis was informed by prior research on mindfulness and chronic pain, our coding process remained inductive and data-driven. As themes coalesced, we identified consistent patterns in how participants described the development of mindfulness skills, which later informed our understanding of participants’ therapeutic progression.

#### PURER Framework of Therapeutic Processing

PURER (Table 1) provides a framework for MORE therapists to engage participants in guided inquiry, referred to as "therapeutic processing" in MORE, about their mindfulness practice (Garland & Howard, 2013). This systematic model of questioning was used after each mindfulness practice during weekly sessions to facilitate participants’ exploration and understanding of their pain experiences. The therapist’s role in this process is to support attention development through phenomenological inquiry about mindfulness experiences, validate participants’ challenges while reinforcing progress, educate participants about normal mind wandering, and encourage participants to continue practicing despite difficulties. In our analysis, we used PURER to provide structure to the analytic approach of in-session qualitative data.

This framework of in-session interviewing provided the structure for data collection and an intimate lens through which to examine participant experiences and progression through the intervention. As session recordings were evaluated, the therapeutic process implemented in the PURER approach emerged as an integral tool supporting the skill development that led to therapeutic change.

Before describing participants’ therapeutic progression, it is important to briefly introduce the specific mindfulness practice taught in MORE. A key practice in MORE is the “mindfulness of pain” technique, which goes beyond general mindful breathing. Participants are taught two complementary attentional strategies: “zooming out” and “zooming in.” Zooming out involves redirecting attention away from the pain (often towards the breath or body as a whole) to reduce emotional reactivity or distress. In contrast, zooming in guides participants to attend directly to the pain itself, observing its sensory features such as intensity, location, texture, and boundaries. Participants learn to decompose pain into component sensations (e.g., heat, tingling, pressure) and to explore surrounding areas for neutral or pleasant contrast. This practice facilitates a shift from affective interpretation to direct sensory awareness, supporting reappraisal and reducing suffering. Both techniques are introduced within the MORE program as therapeutic tools, helping participants build a more adaptive relationship to pain.

## Results

The final sample was predominantly White (81%) and female (70%), with a mean age of 48.59 years and a mean condition duration of 13.72 years. At baseline, participants had an Oswestry Disability Index score of 19.70 and a pain visual analogue scale score of 5.14 (0 = *no pain*; 10 = *most pain*). This subsample was representative of the larger sample of participants included in the primary study results (Wexler et al., 2024). Demographic characteristics can be found in Table 3.

**Table 3:** Demographic characteristics at baseline presented as mean (*SD*) or *n* (%) from Wexler et al. (2024)

Demographics	Total *n* = 37
Sex	
Male	11 (30%)
Female	26 (70%)
Race	
White	30
Black	2
Asian	-
Hispanic/Latino	2
Middle Eastern	1
More than 1 race	1
Other/unknown	1

**Fig. 1:**
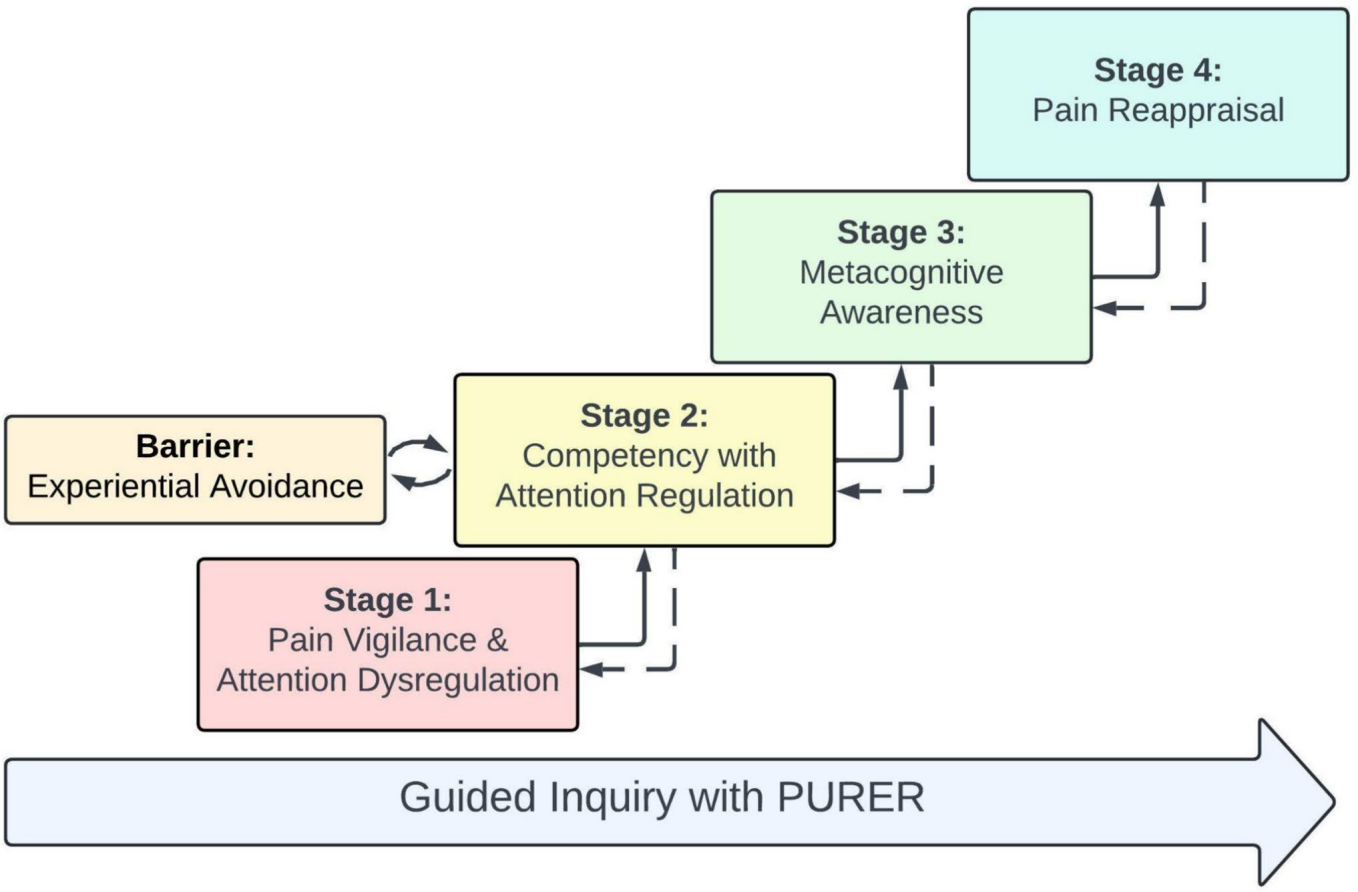
Vigilance-Avoidance Metacognition-Reappraisal (VA-MR) framework. The Phenomenology, Utilization, Reframing, Education, Reinforcement (PURER) framework appeared to support progression through these stages

The qualitative analysis found four distinct stages and one common barrier of mindfulness skill development characterized by participants’ progression through the MORE program: (1) Pain Vigilance and Attention Dysregulation, (2) Attention Regulation and its application to Experiential Avoidance, (3) Metacognitive Awareness, and (4) Pain Reappraisal. We will refer to this process of therapeutic progression as the Vigilance-Avoidance Metacognition-Reappraisal (VA-MR) framework. In addition, we found that the stepwise nature of PURER facilitated progression through VA-MR during the MORE program (Fig. 1). The following quotes describe progression through these stages. It is important to note that, just as with any new skill, individuals can move fluidly between stages of skill development and we observed this in many participants throughout the MORE program.

### Stage 1: Pain Vigilance and Attention Dysregulation

Early sessions in the MORE program focus on building basic mindfulness skills. Here, participants expressed their initial challenges, which generally appear as difficulty maintaining attention — a phenomenon commonly seen in chronic pain patients (Alcon et al., 2025; Battison et al., 2023; Ibrahim & Hefny, 2022). Some participants expressed doubt about their capacity to successfully engage in mindfulness practice:

...maybe I had doubt early on because I have so many attention issues, [I doubted] if I would be able to do this practice well. (Cohort 1, Session 5)

Participants consistently reported difficulties with basic attention regulation in early sessions, particularly around maintaining focus during meditation practices.

The most difficult part... I mean, I feel like it’s this every time, is just sort of staying, like keeping my mind on track with it, just not getting distracted by other things (Cohort 3, Session 3)

MORE therapists deal with this common challenge by reframing the attentional lapse as the practice of mindfulness. This encourages participants to continue working towards the development of mindfulness as a skill by providing reinforcement that they are on the right track.

Physical discomfort also impacted participants’ ability to maintain attention. In particular, participants expressed that their pain made it challenging to be still during meditation:

...if I’m still, my body, the pain in the hips will flare up. So I’ll have to move. And it’s like a butterfly effect with my brain. Once I have that pain and I move, my focus is lost. (Cohort 3, Session 2)

I went to the part that was painful. And I was okay there, but then I think it’s, I don’t know. I had to keep moving. I can’t, I can’t lay still. (Cohort 1, Session 4)

In this first stage, participants demonstrated characteristic patterns of attention dysregulation that manifested as pain vigilance, or an inability to easily move their attention away from their pain experiences (Badiei et al., 2023).

### Stage 2: Competency with Attention Regulation

As participants practiced breath awareness and mindfulness skills emerged, they noted qualitative shifts in their attention regulation abilities over time. The development of these skills was evidenced through multiple participant narratives that demonstrated increasingly sophisticated attentional capabilities:

There’s less fight going on internally as far as the intention goes than it was four weeks ago. It’s almost like a switch now. Whereas before, it was really back and forth, back and forth. And now it’s operating much smoother. (Cohort 1, Session 5)

Looking back, some participants expressed a critical shift from avoidance-based coping strategies to an approach-oriented engagement with pain sensations:

I think for me [before the program], I would just do things to not feel the pain, ya know? I knew it was there all the time, but instead of focusing on it, this helped me go in, relieve it a little more and then let it go. Even though I still have it, but it’s not as bad. (Cohort 1, Session 8)

Over time, participants demonstrated general attention regulation across multiple domains:

When I first started… I was really scattered, and I had a hard time staying in tune with it. And this time, well, I really noticed the whole time, it’s my ability to like jump in and get into that state and stay in that state better than before. (Cohort 1, Session 8)

For example, this participant developed reduced mind wandering, suggesting improved focus; enhanced ability to “jump in” and enter meditative states, indicating improved attentional flexibility; and increased capacity to “stay in that state” and maintain attention over time, reflecting improved attention regulation. While these emerging skills marked important progress, they also introduced a new challenge: some participants used their enhanced attention to avoid pain rather than engage with it.

### Experiential Avoidance

Once participants had developed an initial ability to regulate their attention and a basic competency with mindfulness skills, a tendency emerged in some participants to use these skills for experiential avoidance, avoiding or escaping distressing thoughts or experiences, rather than engagement with pain experiences.

It was hard for me to get past the pain that I have. I kept trying to circle back and try to make it go away, but I was having a really hard time… I’m trying to not think about it. Just think about the breathing and staying with the breathing. (Cohort 1, Session 7)

In some cases, this distancing approach could help provide individuals the time and attention required to approach their discomfort in a therapeutic manner. In general, this kind of experiential distancing is most helpful when participants feel overwhelmed by pain or emotion and lack the resources to engage directly with their discomfort. In these moments, temporarily shifting attention away from the painful sensation may have worked to create a sense of safety (Folkman & Moskowitz, 2004; Sagui-Henson, 2017).

As participants gained basic attention control, many initially employed these skills to avoid pain experiences rather than engage with them mindfully, which manifested as experiential avoidance: “the attempt to avoid internal experiences (e.g., thoughts, feelings, physical sensations) that are experienced as negative (Mohr et al., 2025).” This psychological resistance in fear of the pain experience revealed a complex interplay between attention regulation and pain processing, which manifested as a barrier within the program.

So I’m trying to just listen to you and ignore [the pain]... Ignoring the pain instead of going into the pain. It makes it hurt more…When I ignore the pain, it makes it hurt less. (Cohort 1, Session 6)

Some participants described strong psychological resistance to pain exposure:

I’m just kind of agitated, it’s like my mind is in an agitated state when I’m just laying there. And so part of me feels like, I was dropping in [to the meditation], but I have a lot on my mind. And I notice myself not wanting to do it… feeling very much wanting to distract myself. (Cohort 2, Session 3)

Others noted how when they pay attention to their pain after being attentive to something else, that’s when the pain hurts more.

Now that we’re talking about it again though my leg will start firing. It’s just when I think about it or talk about it too much…I think it’s something I made up but sometimes it will hurt more. (Cohort 2, Session 5)

Some participants even embraced the ability to use mindfulness to re-orient attention away from pain:

It [mindfulness] becomes like a superpower... to fly you somewhere else away from your sorrows and away from the pain. (Cohort 1, Session 7)

These avoidant tendencies led to a common misunderstanding of the therapeutic practice of mindfulness as a process of escape from pain rather than reprocessing and reappraisal of pain sensations, which highlights the importance of skilled guidance and questioning through early stages of mindfulness-based pain management and suggests the need for careful scaffolding of attention regulation skills that work through these challenges.

Collectively, these narratives document a developmental progression in attention regulation characterized by reduced mind wandering, increased volitional control, enhanced metacognitive awareness, and greater efficiency in attentional processes. This stage represents a critical transition point where basic attentional competencies are established, creating the foundation for the more advanced metacognitive skills to emerge.

### Stage 3: Metacognitive Awareness

When participants engaged in continued practice and guidance after mindfulness sessions, they began to display a more nuanced and adaptive capacity to process their pain perceptions by shifting from affective to sensory processing of pain sensations. Through metacognitive awareness of avoidant tendencies, participants were able to begin engaging in healthy forms of pain reappraisal — coming to view pain as an innocuous sensation — and some participants even reported joy in reconnecting with physical sensations:

[It was] quite enjoyable to be able to just kind of soak in and just kind of feel or experience what my body’s experiencing because I’ve really kind of been detached and, more trying to make it go away and not really focusing on feeling it and trying to figure out where it’s at. So it was good to be able to just recognize what my body’s doing. (Cohort 2, Session 4)

Participants also seemed to overcome avoidant tendencies:

I think like the times when I’ve noticed I can relax around it, it helps with the pain. Because then I get to notice it’s shifting. I’m not bracing against it or like trying to avoid it. (Cohort 2, Session 1)

The therapeutic benefit of metacognitive awareness led to clear shifts in participants’ relationship with pain and improved physical function. A key shift involved participants developing the ability to observe their own mental processes and the impact of these processes on physical sensations.

### Stage 4: Pain Reappraisal

Participants adapted their relationship with pain via the metacognitive process of reappraising present moment experiences and reinterpreting signals in the context of pain. When participants were able to bring mindful awareness to areas of their body with pain through the “mindfulness of pain” technique taught in MORE, it enabled them to shift from affective to sensory processing of the pain experience (Garland et al., 2023), and they expressed a shift in their pain experience:

The more I focused on the breath at that specific point it just seemed like the intensity of the pain decreased into a cool feeling... to where I could kind of calm things down by utilizing the breath. (Cohort 4, Session 3)

When I went into the pain part of it, it was very jagged and sharp and hot and red... as I breathed more into it, it got less sharp, more of a dull (Cohort 1, Session 7)

We observed that some participants used reappraisal to change their relationship to pain entirely and to distinguish their identity from that of their pain experience:

I have to redefine my definition of pain... that’s kind of what I’ve been set out to do is to redefine my definition of pain and not let it define me and control me and, you know, keep me just useless. (Cohort 1, Session 6)

Participants reported fundamental changes in how they experienced and related to pain:

It feels like a sense of freedom really, you know, before you feel almost like you are getting a life sentence, like it’s not going to end. Yeah, it’s really, I feel a little bit like I have a new lease on life, endless of a thought of a life sentence, you know, which is really freeing, which is awesome. And I’m ready to like mentally I’m ready to take off and go running not quite physically though. So like my new lease on life is pushing me through a little bit more for sure, but then maybe at the same time pushing me a little too far at times. (Cohort 1, Session 8)

[I’m] noticing the way that I’m noticing pain... I feel like my relationship to it is changing. Which is great... I noticed that like today, I was walking. And, I don’t remember the last time I walked and like, didn’t immediately notice pain. I walked out of my house. And, and I think I had gone like a block and a half. And I was like, oh my gosh, like I don’t, I’m not experiencing the same kind of pain. (Cohort 2, Session 3)

### The PURER Framework Facilitates Skill Development in Metacognitive Awareness

We observed that skillful and systematic implementation of MORE’s guided inquiry process, PURER, supported participants in developing an adaptive relationship to their pain. Rather than simply teaching mindfulness techniques, therapists used PURER to help participants navigate challenges and develop increasingly nuanced relationships with pain experiences. In early stages, where participants struggled with basic attention regulation, therapists asked participants to describe their phenomenology, or moment-to-moment experience, to help participants investigate their meditation. Once participants had discussed what occurred for them during the practice session, therapists could use the participant’s experience as an example of an important concept being taught. This example, from the first session of the program, describes a participant who was prompted to report novel insights about their anticipations of the pain experience:

Therapist: “I was wondering if maybe you could zoom into a particular part of the meditation when that was happening and tell me how that happened with you and your mind and your breath?”

Participant: “Every part of the body that we went to, my body, like, I felt more pain... it wasn’t like constant, and it wasn’t like... excruciating. it was just like, ‘Oh, you’re noticing me and here I am’... I noticed like tension right here as you were approaching that... and I was like, that’s anticipation... my brain is already deciding that it’s kind of hurt before I even get there. So I actually tried to relax right here because I felt myself doing that. And I feel like that actually helped.” (Cohort 2, Session 1)

As participants began showing capacity for metacognitive awareness, therapists used phenomenological inquiry to deepen this emerging skill. The therapist reinforced this metacognitive observation while helping to utilize it for pain management.

Therapist: “What did you enjoy about today’s practice?”

Participant: “I’m really relaxed. A lot less pain.”

Therapist: “Good, that’s fantastic. When did that start for you in the meditation, [participant name]?”

Participant: “Um, I went deep in, came back out, and went deep in. Each time it was a little less.”

Therapist: “So you found that every time you went back to observe the pain, there was a little bit less there to notice?”

Participant: “Mmhm. Yeah. It got softer and softer. Not as deep.”

Therapist: “That’s fantastic. I want to also take some time today, as we are debriefing that experience to focus or reflect on how things have changed over the course of these 8 weeks, 9 weeks now. How does that compare to some of the earlier experiences you had in the program? Do you find that the pain went away quicker? Or did it linger about as long as it used to but you were just more aware of it?”

Participant: “I think for me, I would just do things to not feel the pain, ya know? I knew it was there all the time, but instead of focusing on it, this helped me go in, relieve it a little more and then let it go. Even though I still have it, but its not as bad.” (Cohort 1, Session 8)

In general, it appeared that the value of PURER is engrained in how its components worked together to support mindfulness skill development. As participants developed basic attention skills, but showed avoidance tendencies, therapists used phenomenological inquiry to help participants recognize and investigate their avoidance. Rather than criticizing avoidance, therapists used reframing and education to help participants understand its limitations while reinforcing their growing skillset. Finally, when mindfulness strategies were successfully utilized, therapists provided positive reinforcement about participants’ experiences:

Therapist: “Do you remember during the meditation when you felt the calm start?”

Participant: “You were talking about feeling your breath… I started to do that and just pay attention to what it was doing.”

Therapist: “Do you remember what happened next?”

Participant: “Yeah. I just feel like I could just like stay there… When things you know annoy me.”

Therapist: “That is practicing mindfulness and applying it.”

The systematic nature of PURER helped ensure key therapeutic elements were consistently present, while its flexibility allowed adaptation to participants’ current stage and specific challenges. The framework particularly excelled at helping participants move from avoidance to engagement through careful phenomenological inquiry, strategic reframing, and consistent reinforcement of pain education in the MORE program. This structured and responsive guidance appears crucial for realizing therapeutic benefits from mindfulness-based pain management programs such as MORE.

## Discussion

By qualitatively analyzing audio recordings of MORE sessions, this study offers a unique perspective on the lived experiences of individuals with LRP as they begin a new mindfulness practice. This approach allowed us to capture the nuances of participants’ evolving relationship with pain in real time, providing valuable insights into the therapeutic process facilitated by MORE. These findings highlight a progression in how participants develop adaptive relationships with pain through mindfulness practice. An important finding in this work was that the process of mindfulness skill-building was not unidirectional. Participants appeared to move between stages of skill development from week to week and even from meditation to meditation within a single weekly session. Participants’ pain experiences in meditation appear to lie along a spectrum on which skill development occurs over time, but challenging experiences, such as difficulty with attention regulation, can continue to occur after weeks of practice. Our findings extend previous research by illuminating both the challenges and transformative processes that can occur during mindfulness-based pain management interventions.

### Key Findings and Theoretical Implications

It is important to note that participants often moved fluidly between the non-linear stages of therapeutic progression in VA-MR. In addition, our findings reveal an important paradox regarding the relationship between attention regulation strategies and functional outcomes in early mindfulness skill development. While participants successfully developed basic attention regulation abilities, they often deployed these skills for experiential avoidance rather than engagement with the experience of pain. This pattern aligns with Hayes’ theory of experiential avoidance (Hayes et al., 1996; Hayes-Skelton & Eustis, 2020). Importantly, we do not interpret this early use of attentional regulation as a therapeutic endpoint of MORE or as a form of encouraged “refocusing away” from pain. Rather, our data suggest that participants initially misapplied their emerging attentional skills towards avoidance, which is consistent with early stages of mindfulness learning. In contrast to avoidance, the intended trajectory within mindfulness practice, and within MORE specifically, involves bringing attention towards sensory experience with an attitude of equanimity and non-judgment. This stance supports decreased self-referential processing, greater cognitive flexibility, and ultimately the reappraisal of pain sensations (Riegner et al., 2023). The PURER framework appears to play a critical role in helping participants shift from avoidance-based to equanimity-based engagement. Thus, the avoidance barrier we observed reflects a developmental waypoint rather than a mechanism reinforced by the intervention.

### The Dichotomy of Pain and Disability

Our findings also highlight a critical observation from the parent clinical trial: while MORE produced a significant reduction in pain intensity, these improvements did not translate to corresponding reductions in disability (Wexler et al., 2024). The discrepancy between changes in pain intensity and disability presents an important theoretical and clinical question that our qualitative analysis begins to address. We observed participants demonstrating two distinct approaches for attending to pain management through mindfulness: avoidance-based attention regulation and metacognitive awareness–based attention regulation. This dichotomy warrants critical examination within the context of disability outcomes.

Participants in the avoidance stage successfully employed attention regulation techniques to reduce pain intensity, as evidenced by statements like: “When I ignore the pain, it makes it hurt less” (Cohort 1, Session 6). However, this strategy may create a therapeutic “ceiling” regarding improvements in disability. While momentary pain reduction occurs, participants may remain unable to effectively direct their attention towards the affected areas when necessary, potentially maintaining disability levels despite a reduction in pain (Suso-Ribera et al., 2019; Zale et al., 2013).

Participants who developed metacognitive awareness often reported awareness of pain sensations but demonstrated improvements in disability. One participant noted, “I walked out of my house… and I think I had gone a block and a half. And I was like, ‘Oh my gosh, like I don’t, I’m not experiencing the same kind of pain!’” (Cohort 2, Session 3). This suggests that while the sensory dimension of pain may persist, one’s affective response to pain and one’s relationship to the pain sensation is malleable, resulting in reduced disability. In MORE, patients are taught unique mindful breathing and body scan meditations designed to deconstruct the pain experience into its constituent sensations (e.g., heat, tightness, tingling), as well as to increase awareness of the center, edges, and permeability (versus solidity) of these sensations, and any adjacent pleasant sensations. This practice of cultivating mindful awareness of pain may decrease emotional reactivity to pain and thereby decrease pain intensity. Moreover, this practice may disentangle self-referential processes from pain-related sensory input, a therapeutic approach supported by previous neurobiological research on the relationship between pain and participants’ sense of self (Riegner et al., 2023; Zeidan et al., 2015, 2019).

This observed dichotomy has significant implications for understanding therapeutic mechanisms in MBIs. Although initially learning to orient attention away from pain via mindful breathing may produce analgesic effects (Zeidan et al., 2015), the development of pain *tolerance* through directing meta-awareness towards pain — rather than experiential avoidance — may represent a more sustainable pathway to improvements in pain-related disability. Our findings suggest that the ability to direct attention *towards* pain when necessary, while maintaining an emotional equilibrium, typically referred to as “equanimity” within the context of meditation, may be important for achieving reductions in disability. These observations help contextualize the relationship between pain intensity and disability measured in the parent trial (Wexler et al., 2024) in which pain intensity was reduced while disability was not. However, it should be noted that prior full-scale RCTs have demonstrated that MORE significantly decreases pain-related functional interference, including measures of physical, social, and occupational function (Garland, 2024; Garland et al., 2014, 2022). These conflicting results suggest that further investigation into pain-related attention is warranted.

More generally, our findings point to the relevance of interoceptive awareness in chronic pain (Horsburgh et al., 2024). Because avoidance can erode contact with bodily sensations and reduce interoceptive awareness, MBIs may benefit from incorporating brief practices that target interoceptive awareness directly to help restore adaptive engagement with internal experiences. In addition, evidence on mindfulness-based interoceptive exposure suggests that equanimity towards pain sensations may function as a dose-dependent mechanism. In this study, participants practiced brief, repeated exposures to pain sensations with equanimity and non-identification each time pain intensified during daily life, and therapeutic effects appeared to rely on this continual practice (B. Cayoun & Shires, 2020a). In contrast, our qualitative data only reflect in-session experiences; therefore, it is possible that insufficient or inconsistent practice limited participants’ ability to integrate equanimity-based experiences with the experience of pain — a process that may be necessary for translating reductions in pain intensity into improvements in disability. Future work should examine how practice dosage shapes the development of equanimity, meta-awareness, and functional outcomes in MORE.

### Phenomenological Inquiry Facilitates Metacognitive Development

The PURER framework appeared to be particularly valuable in our study in facilitating the transition from avoidance-based pain management to metacognitive awareness–based pain management. PURER emerged as crucial in facilitating the transition from avoidance to engagement with the experience of pain. This structured approach to inquiry helped participants move beyond initial avoidance tendencies towards a therapeutic engagement with their pain experience: “noticing the way that I’m noticing pain… I feel like my relationship to it is changing” (Cohort 2 Session 3). This transformation suggests that therapist guidance through the PURER framework may be essential for realizing therapeutic benefits from MORE. In addition, the valuable role of the therapist highlights the importance of maintaining fidelity to the MORE program when delivering the intervention (Hanley et al., 2020).

Finally, participants developed an increasing metacognitive awareness of their experience of pain. The progression from basic attention regulation to complex reappraisal is evident in multiple participant narratives. This development of metacognitive skills appears to be a key mechanism in transforming participants’ relationship with pain and is taught to MORE participants using the skill of mindful reappraisal of pain.

### Clinical Implications

Clinicians should anticipate that patients with LRP may initially use mindfulness skills for experiential avoidance of their pain experience. The data suggest that acknowledging and working skillfully with avoidance, rather than treating it as failure, may be crucial for therapeutic progress. Early identification of avoidance patterns can help therapists guide participants towards greater engagement with their pain experience.

Our findings also highlight the importance of carefully scaffolding the transition from basic attention training to mindful exposure to pain. The challenges documented in our analysis, such as “I just had a hard time getting settled… I couldn’t get comfortable” (Cohort 1 Session 4), suggest the need for a gradual progression and robust support during this phase.

Finally, the success of the PURER model in facilitating change suggests the importance of maintaining fidelity to this structured approach to therapeutic processing. Therapists should be trained to skillfully implement each component of PURER, using them systematically to support participants’ progress through MORE. As facilitators learn to deliver MORE and apply the PURER model, the MORE Fidelity Measure should be used to ensure therapist competence and adherence to the model (Hanley & Garland, 2021).

### Interpretation of Findings in Relation to Existing Literature

Our results align with and extend previous research on the use of MBIs for chronic pain. The shifts in pain perception reported by our participants, characterized by increased metacognitive awareness, reappraisal of pain sensations, and a move towards acceptance, support the notion that mindfulness practices can alter the cognitive and affective dimensions of pain, even when sensory aspects persist (Kabat-Zinn, 1982; Reigner et al., (Riegner, et al., 2025)). In addition, the present results provide a detailed account of how metacognitive awareness develops over time and its specific application to LRP symptoms. Participants were able to observe their pain with greater detachment, a key psychological mechanism of mindfulness interventions called decentering (Hanley et al., 2020; Hick & Chan, 2010).

The process of mindful reappraisal of pain sensations (Garland et al., 2023), observed in our study, extends beyond simple distraction techniques often used in pain management programs. Instead, it involves a sophisticated re-evaluation of the pain experience, consistent with previous research (Ashar et al., 2022; Schütze et al., 2010). In addition, the observed shift from experiential avoidance to greater acceptance of pain aligns with the psychological flexibility model underlying acceptance and commitment therapy (ACT) for chronic pain (McCracken et al., 2007; Vowles et al., 2015). However, the pain-relieving effects of MORE have been shown to be statistically mediated by the reinterpretation of pain as an innocuous sensation (Garland et al., 2014, 2023), demonstrating that MORE operates through mechanisms other than acceptance through its integration of mindfulness meditation, reappraisal, and savoring practices.

### Limitations and Future Research

A key strength and novel aspect of our study is the use of session recordings for qualitative analysis. This approach allowed us to capture the dynamic, moment-to-moment experiences of participants as they engaged with the MORE program, providing a level of detail and immediacy often missing from retrospective interviews or quantitative measures. The analysis of session recordings revealed the nuanced ways in which the PURER model guided participants’ exploration and processing of their pain experiences. These findings contribute to our understanding of the specific components of MORE that may drive its therapeutic effects, addressing calls in the literature for greater specificity in understanding the active ingredients of MBIs (Mohr et al., 2025; Van Gordon, 2017). Our study also provides insights into the challenges and barriers faced by individuals with LRP in engaging with mindfulness practices. These findings can inform the refinement of MBIs to better address the specific needs of this population, potentially improving adherence and outcomes. Finally, our qualitative findings are corroborated by quantitative data from our previous publications indicating that participants experienced increases in scores on both the Mindful Reappraisal of Pain Sensations scale and the Five Facet Mindfulness Questionnaire — a measure of trait mindfulness (62.79% and 8.41% increases, respectively) (Wexler et al., 2024).

While our study provides valuable insights, it has important limitations. First, our analysis focused on session recordings, where the virtual delivery format may have influenced participant experiences and therapist–participant interactions. Future research should examine whether similar progression patterns emerge within in-person settings. Second, this study only examined participant experiences with meditation during the MORE program and did not collect data on how participant mindfulness experiences may have changed after completing the intervention. Third, this study primarily included Caucasian women, which may limit the generalizability of our findings to other populations. As we have discussed previously, national surveys of complementary and integrative health have shown higher utilization among women and white adults than other demographic subgroups (Alwhaibi & Sambamoorthi, 2016; Keshet & Simchai, 2014; Wexler et al., 2025). Fourth, while our analysis identified clear progression patterns, individual variations in this progression warrant further investigation. Due to the group design of this study, we were not able to identify specific participants on the audio recordings and track their progression over the course of the program. Rather, we were identifying shifts in an entire cohort’s understanding of MORE concepts from week to week via longitudinal qualitative analysis (Grossoehme & Lipstein, 2016). Finally, in situating these findings within the broader contemplative context, it is important to note that traditional mindfulness practice is embedded within a larger system that includes ethical foundations, insight, and the cultivation of wisdom. Modern MBIs, including MORE, draw selectively from these traditions to target specific psychological mechanisms relevant to chronic pain. Acknowledging this distinction clarifies that the therapeutic processes identified here reflect particular components of a wider contemplative framework rather than the full scope of classical mindfulness training.

The four-stage progression model identified in this study may serve as a foundation for future efforts to track therapeutic progress more efficiently. With recent advances in artificial intelligence, researchers are beginning to explore whether large language models can help analyze qualitative data, such as session transcripts, in a more automated way. Early studies have shown that this approach is feasible (Lennon et al., 2021). Building on these developments, future research could use clearly defined therapeutic stages, like those described here, to support structured, theory-driven analysis of participant experiences. This could make it easier to refine and improve mindfulness-based interventions, even in small studies.

Our findings suggest several promising directions for future research. The stages of skill development that we identified may be associated with important clinical outcomes, such as kinesiophobia or disability. Future studies might also examine factors that facilitate or impede progression through these stages. Assessing these stages throughout intervention delivery, as is done in iterative user-/human-centered design (Alwashmi et al., 2019), could allow the development of more personalized teaching and therapy strategies. Given the central role of therapist guidance in navigating experiential avoidance and supporting progression through the identified stages, it is important to emphasize that the effective delivery of PURER requires a high level of clinical skill and training. Studies that aim to build on this work should consider investigating strategies to mitigate the tendency towards experiential avoidance early in the program and attempt to assess a participant’s progression through the various stages of mindfulness skill development in real time.

### Conclusion

This qualitative study identified a four-stage progression in mindfulness skill development among individuals with lumbosacral radicular pain: attention dysregulation, competency with attention regulation, metacognitive awareness, and pain reappraisal. These stages provide a framework for understanding common challenges and therapeutic opportunities in mindfulness-based pain interventions. Crucially, the PURER model of therapeutic processing facilitated this progression, helping participants move from avoidance towards adaptive engagement with pain. These findings offer a clinically actionable model for tailoring instruction, reinforcing the importance of therapist skill and program fidelity. As nonpharmacologic strategies for chronic pain evolve, incorporating structured, stage-informed guidance into interventions like MORE may improve outcomes for patients with LRP and related conditions.

## Data Availability

The data that support the findings of this study are available from the corresponding author, RSW, upon reasonable request.

## Declarations

### Use of Artificial Intelligence

Artificial intelligence was used for editing the manuscript to improve conceptual clarity.
